# Healthy weight services in England before, during and after pregnancy: a mixed methods approach

**DOI:** 10.1186/s12913-020-05440-x

**Published:** 2020-06-22

**Authors:** Frankie Fair, Katie Marvin-Dowle, Madelynne Arden, Hora Soltani

**Affiliations:** 1 College of Health, Wellbeing and Life Sciences, Collegiate Campus - Sheffield Hallam University, 34 Collegiate Crescent, Sheffield, S10 2BP UK; 2 Departmental Research & Scholarship Lead, Department of Psychology, Sociology & Politics, Collegiate Campus - Sheffield Hallam University, Sheffield, S10 2BQ UK

**Keywords:** Healthy weight, Gestational weight gain, Maternal obesity, Service provision

## Abstract

**Background:**

Maternal overweight and obesity are associated with numerous adverse outcomes including higher rates of maternal and infant mortality and morbidity. Overweight and obesity before, during and after pregnancy are therefore a significant public health priority in England. This project explored and mapped healthy weight service availability at different stages of the childbearing cycle.

**Methods:**

A mixed methods approach included a questionnaire-based survey disseminated through Local Maternity Systems and semi-structured interviews or focus groups with providers and commissioners. Current maternal weight service provision was explored along with some of the barriers and facilitators for providing, delivering and accessing healthy weight services. Descriptive statistics were reported for quantitative data and content analysis was used for thematic reporting of qualitative data.

**Results:**

A total of 88 participants responded to the survey. All services were offered most frequently during pregnancy; with healthy eating and/or weight management services offered more often than physical activity services. Few services were targeted specifically at women with a raised body mass index. There was a high degree of inconsistency of service provision in different geographical areas.

Several themes were identified from qualitative data including “equity and variation in service provision”, “need for rigorous evaluation”, “facilitators” to encourage better access or more effective service provision, including prioritisation, a change in focus and co-design of services, “barriers” encountered including financial and time obstacles, poor communication and insufficiently clear strategic national guidance and “the need for additional support”.

**Conclusions:**

There is a need to reduce geographical variation in services and the potential health inequalities that this may cause. Improving services for women with a raised body mass index as well as services which encourage physical activity require additional emphasis. There is a need for more robust evaluation of services to ensure they are fit for purpose. An urgent need for clear national guidance so that healthcare providers can more effectively assist mothers achieve a healthy weight gain was identified. Commissioners should consider implementing strategies to reduce the barriers of access identified such as childcare, transport, location and making services free at the point of use.

## Background

Obesity and overweight (Body Mass Index (BMI) ≥25 kg/m^2^) affect more than 60% of the adult population [1]; with data from 37 maternity units showing the rate of first trimester maternal obesity has more than doubled over the previous 2 decades [2]. Managing the consequences of obesity is estimated to cost the National Health Service (NHS) £27 billion per year [3]. Childbearing contributes to the rise of overweight and obesity in women [4]. Raised BMI is associated with increased short-and long-term adverse outcomes for mothers such as increased risk of maternal mortality, pregnancy induced hypertension, gestational diabetes, primary postpartum haemorrhage and interventional birth [5]. For babies, there are additional risks of stillbirth, large for gestational age, admission to neonatal units and neonatal mortality [6–11].

The promotion of healthy lifestyle and healthy weight before, during and after pregnancy have been suggested to reduce the risk of pregnancy and birth complications as well as to minimise the risk of obesity development and metabolic diseases such as type 2 diabetes in the long term [12–14]. Implementing preventative strategies that provide opportunities to enhance lifestyle choices throughout preconception, pregnancy and the postpartum with the aim of reducing the burden of maternal obesity and its associated complications have been identified as top priorities [15]. However, little is known about the pattern of service provision to support women and families to enhance their lifestyle and promote healthy weight gain at such an important life stage.

In 2015 the United Kingdom (UK) government announced its ambition to halve rates of stillbirth, neonatal death and maternal death by 2030 [16]. In 2016 ‘Better Births’ set out the vision for safer, more personalised, kinder, professional, and more family-friendly maternity services [16]. While NHS England lead the overall programme, Public Health England (PHE) leads the implementation of Workstream 9 ‘Improving prevention and population health’ within the Better Births recommendations [15]. Local Maternity Systems (LMS) have been established to drive this transformation and are responsible for developing and implementing a local vision for transforming maternity services by 2020/2021. In total forty-four LMS have been established across England which include both providers and commissioners operating together to ensure that women and families are able to access the services they need in a timely manner.

This study aimed to explore current service provision of maternal healthy weight services in England through a survey of LMS representatives and to determine the perspectives of key stakeholders on variation in service provision and related barriers and facilitators locally.

## Method

A mixed methods approach was undertaken. It is recognised that combining different research methodologies in mixed methods research can provide better understanding of research questions through triangulation of results collected from different sources [17]. An online questionnaire was distributed and analysed. The results of this were utilised to develop a semi-structured interview schedule which was used to gather further data from key stakeholders.

### Online survey

Using an online Survey Monkey questionnaire, we explored commissioners’ and providers of healthcare services’ views to establish the various range and type of services available to support women in achieving a healthy weight and lifestyle prior to pregnancy, during pregnancy or up to 1 year postpartum (see Additional file 1). The survey included fixed response and open-ended questions around services available for all women with regards to healthy eating (services specifically aimed at promoting a healthy diet), physical activity (services aimed at encouraging exercise) and weight management (services aimed at weight management which could address diet, exercise or psychosocial issues). The survey also asked about any services available for women with a raised BMI (≥25 kg/m^2^). Where services were provided, respondents were asked if the service had been evaluated in any way, this could include local audit or formal external evaluation. Respondents were also asked if the services provided, were for the women only or incorporated other family members into the service and whether service users had been involved when developing the service.

The survey was disseminated through the national maternity transformation programme network to all 44 LMS. The questionnaire was initially sent out on 12th March 2018, followed by a reminder on 24th April 2018. Further requests were sent to LMS Chairs within regions where no initial responses were received.

### Semi-structured interviews

Individual interviews or focus groups were undertaken with a purposive sample of key stakeholders within one region of England. These stakeholders included providers and commissioners of maternity, public health, sexual health, contraceptive and health visiting services. A semi-structured interview schedule informed by the survey results was developed (see Additional file 2). This covered services currently provided, services that respondents would like to see provided and factors interviewees felt were facilitators or barriers to service provision or access.

### Data analysis

Descriptive statistics were reported for the quantitative component of the survey and simple content analysis undertaken on the open-ended responses within the survey.

All interviews and focus groups were audio recorded and transcribed verbatim. Themes within responses were identified using a simple content analysis. Transcripts were initially read to ensure familiarity with the data. Interviewees’ responses were then coded inductively by two researchers to enhance trustworthiness of the findings. Similar and disparate codes were developed into themes. Two researchers undertook the analysis with the final themes agreed through discussion.

## Results - online questionnaire

### Characteristics of respondents

Responses were received from 88 people of whom 39 (44.3%) were commissioners, 43 (48.9%) were providers, 1 (1.1%) was a specialist, 1 (1.1%) worked as both a commissioner and provider and four (4.5%) did not answer.

**Fig. 1:**
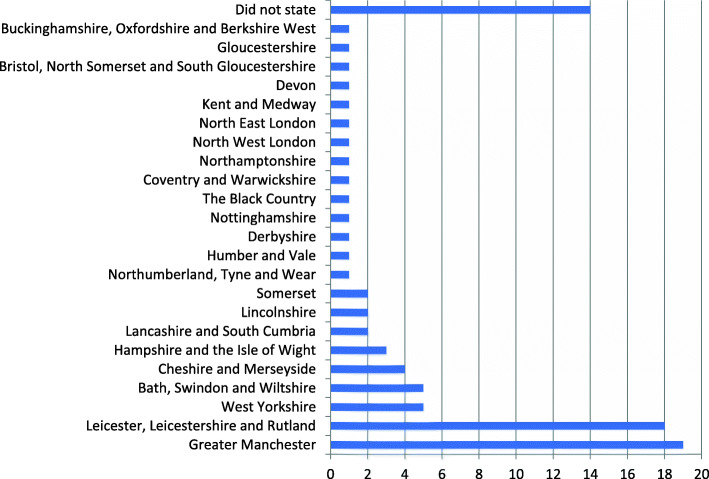
Survey responses from the different Local Maternity Systems (LMSs) (*n* = 88)

Figure 1 shows the LMS distribution of respondents. A response was received from 23 of the 44 LMS (52.3%) within England. Of the fourteen respondents who did not report which LMS they were from, 11 took no further part in the survey.

### Service provision for women before, during or after pregnancy

**Fig. 2:**
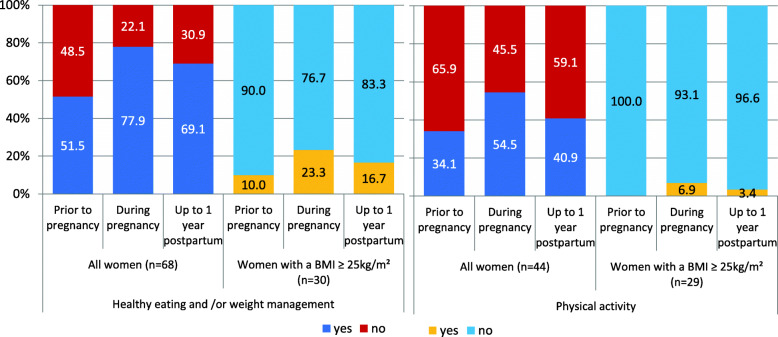
Service availability to all women and women with a raised BMI at different childbearing stages

A total of 68 participants answered questions about service availability for all women promoting healthy eating and/or weight management and 44 responded to questions about physical activity. Both types of services were offered most frequently during pregnancy and the time that services were reported to be offered least was prior to pregnancy. Healthy eating and/or weight management services were offered more often than physical activity services at all stages of the childbearing cycle (See Fig. 2).

Additional service provision for women with a BMI ≥ 25 kg/m^2^ was low at all stages of the childbearing cycle (Fig. 2); with very few respondents reporting additional services (only 7 healthy eating and/or weight management services and 3 physical activity services). Similarly to services for all women, most services were provided during pregnancy.

### Geographical distribution of services

Service availability varied between different LMSs, with some LMSs reporting no services during the childbearing cycle, some reporting provision across all stages and some at one or two of the different stages only - prior to pregnancy, during pregnancy or postpartum.

In all LMS where there were multiple respondents it was noted that the services reported varied between respondents. This could be from no service availability to availability across all stages of the childbearing cycle. It was unclear within our survey whether this was due to service availability awareness differing between respondents or whether there were differences in service availability in different NHS Trusts/ clinical commissioning groups within each LMS.

### Service components

Services provided varied. They could include basic information provision such as the Eat Well Plate [18], safe exercise in pregnancy guidelines or the physical activity in pregnancy infographic [19] given by professionals such as midwives or health visitors. Some areas provided information about bespoke programmes that women could access such as aquanatal classes, Cook it Programmes, leisure centre partnerships or park walks. A few respondents reported the availability of dietician referral or could refer women to specific services such as HENRY (Health, Exercise and Nutrition for the Really Young) [20].

#### Family approach to the services

**Fig. 3:**
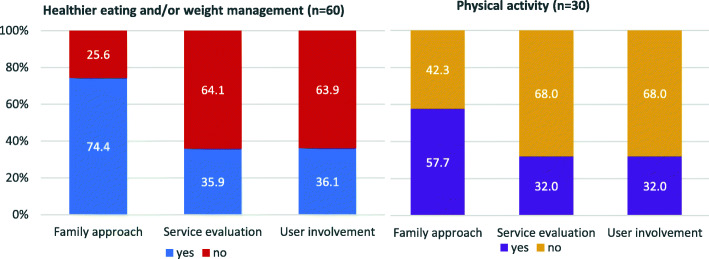
Services reporting that they had been evaluated, used a family approach or had involved service users

A family approach was reported to be taken in 74.4% of healthy eating / weight management services and in 57.7% of physical activity services (Fig. 3). Family approaches included other family members being incorporated into the service, whole family activities for example cookery courses or proactive onward referral for family members. Some respondents also felt that changing the mothers’ lifestyle would impact the long-term health of all family members.

#### Evaluation of the services

Only 35.9% of services encouraging healthy eating or weight management and 32.0% promoting physical activity reported to have been evaluated (Fig. 3). Evaluation was mainly through service user feedback or internal audit. Only four services had been independently evaluated, either by an independent University or national evaluation of services such as Slimming World. None of the services for women with a raised BMI reported they had been formally evaluated.

#### Service user involvement in services

Over a third of respondents reported service user involvement (Fig. 3). This included co-creation of the service, service development through user feedback or service user involvement on research steering groups.

Results of the survey are also summarised on the infographic in Additional file 3.

## Results - semi-structured interviews

### Characteristics of participants

Thirteen participants undertook semi-structured individual interviews (*n* = 6) or focus groups (two focus groups with 2 and 5 participants respectively). Participants had a wide geographical distribution within two LMS and varied professional roles including council public health workers (*n* = 7), specialist or consultant midwives whose role included public health (*n* = 4), a health visitor (*n* = 1) and a council-sports partnership worker (*n* = 1).

### Service provision description from interview participants’ perspectives

#### Services provided for all women

Services provided for all women were similar to those described within survey responses, including information provision by midwives or health visitors (*n* = 8), HENRY and it’s follow on initiatives (*n* = 5) or other initiatives to promote healthy eating and physical activity (*n* = 2), exercise groups such as aquanatal or buggy fit (*n* = 6) and referral to local leisure centres (*n* = 5). Some interviewees also discussed additional provision not mentioned by survey respondents including links with schools and colleges to provide pre-conception education (*n* = 1), achieving Baby Friendly Initiative [21] accreditation or providing breastfeeding support (*n* = 2), ‘Fitmums and Friends’ and ‘This Girl Can’ - campaigns to promote women to exercise (*n* = 1), initiatives targeting healthy food provision options in vending machine (*n* = 2) and the Promotional Guide Tool [22] used by health visitors to promote conversations with women regarding lifestyle (*n* = 1).

#### Service provision for women with a raised body mass index

Similarly to the survey, there were few services specifically for women with a raised BMI. Some had consciously move away from targeted services to avoid stigmatisation, while others had experienced decommissioning of services due to funding issues (*n* = 3).

### Thematic representation of the interview findings

**Fig. 4:**
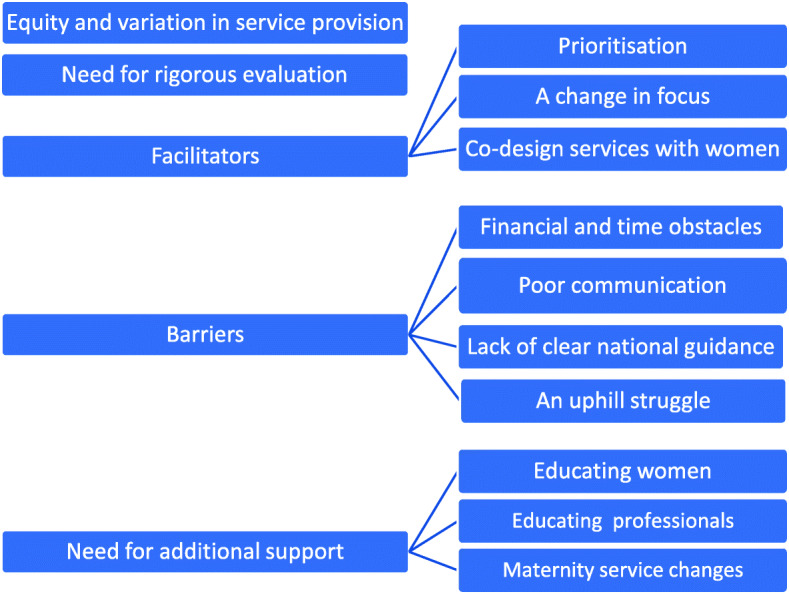
Thematic representation of key stakeholders’ views on maternal weight management service provision

The themes and subthemes identified from qualitative data are presented in Fig. 4 and discussed in detail below.

### Equity and variation in service provision

In line with survey results, geographical variation in service provision was noted both between areas and within areas. In one area women within the same NHS Trust for maternity care could have access to different services dependent on where they lived, due to councils which commissioned services having different boundaries to NHS Trusts.

*“I see women on a Wednesday morning and I can only say you can go to Slimming World if you want but you’ll have to pay for it yourself and then I see ladies on a Thursday morning and I say this is the lovely XXXX [*who runs the free local maternity programme for women with a raised BMI*]. It’s just unequal”* Participant 2.

### Need for rigorous evaluation

Evaluation of services was universally poor. The exception was the national HENRY programme which had been independently evaluated [23]. Any local evaluation described was by monitoring attendance or asking women for feedback, with changes incorporated into the service in response to any issues raised.

Respondents felt limited specific guidance on acceptable weight gain during pregnancy as standards to measure services against hindered evaluation. One service however used the Centre for Maternal and Child Enquires maternal obesity report [7] as a UK baseline for outcomes such as Caesarean delivery, induction of labour and preterm birth, with which to compare their service outcomes.

*“I think it’s very difficult in pregnancy to evaluate it properly because we don’t have a true reference range for what is a healthy weight gain.”* Participant 7.

### Facilitators for weight management service provision and access

Suggestions made by interviewees to encourage better access or more effective service provision, included prioritisation, a change in focus and co-design of services.

#### Prioritisation

Prioritisation of the first 1000 days by one council had allowed additional funding to be attained for high priority areas, to commission additional services. The high-level initiative Healthy Weight Declaration was also seen to support local government to develop and implement policies around healthy weight for example changing food provision at community cafes or parenting groups [24].

“*So there’s things like the healthy weight declaration … that’s something that we’re trying to take on board here and it’s very much about changing the culture around food, activity and the environment essentially. So you know the food that’s provided in hospital vending machines or gym vending machines, so that’s been challenged so that healthier food is provided*” Participant 1.

#### A change in focus

There was a shift from focussing exclusively on weight onto healthy eating and activity. The importance of establishing the individual’s current understanding of weight management and encouraging women to identify their own goals was also considered to be important, rather than imposing the provider’s ideas.

*“on a general level I think the focus needs to move away from weight and more onto health of eating well and moving more and shifting the culture in that way”* Participant 1.

#### Co-design of services with women

Clear communication with service users to ensure appropriate services are commissioned was considered key to ensure access. Providing free services, in places with good transport links, on-site childcare, high visibility through good marketing and de-centralised services to reduce the distances women have to travel were all considered vital to improve service accessibility.

*“being commissioned appropriately with discussion with services and with women ... because sometimes I think when services are being commissioned they don’t think about the people who’re actually using them, so it’s so important asking the right women”* Participant 5.

Providing services for all women was felt to increase service uptake, by preventing mothers with a raised BMI feeling stigmatised. This also made sense to providers given the increasing population levels of overweight and poor diet and the lower cost associated with embedding a universal service into existing provision, rather than paying for an additional service.

*“Regardless of people’s weight, we know that people have poor diets. I think whenever we talk about targeted interventions, we always just come back to we might as well do it universally”* Participant 13.

### Barriers to weight management service provision and access

Numerous barriers that inhibited provision, promotion and access to healthy lifestyle services were identified.

#### Financial and time obstacles

Lack of money was the biggest obstacle reported. It prevented services being commissioned, prevented investment in services and inhibited service delivery. Rural areas were especially hard hit as running services in areas where pregnant women were very dispersed was not cost effective. Money restrictions also equated to time restrictions within appointments, meaning discussions around healthy eating or referral to available services were always a low priority for midwives needing to address many other topics. The proper evaluation of services was also impeded by lack of money.

*“It’s a difficult one because the council don’t have the budget anymore.”* Participant 4.

The constant cycle of commissioning, decommissioning, revamping and re-commissioning of services as funding was available made it difficult for practitioners to stay informed with service availability.

*“I know staff find it so frustrating when things are coming and going because they say you know it’s fantastic this and the next minute you’re putting a message saying actually the services have stopped.”* Participant 5.

#### Poor communication

Commissioners reported difficulties in evaluating services as they were unable to assess the quality of frontline staff conversations. They also reported frustration at not receiving information such as attendance when it was requested from providers, so did not know whether further promotion of services was required.

*“They [midwives] tend to record that a discussion has to take place, but the quality of the discussion could vary so I could say, well you know you are pregnant, now you must eat well and exercise and then tick my box, or ... it could be a bit more of an open discussion with a bit more quality to it”* Participant 11.

#### Lack of clear strategic national guidance

Limited evidence on interventions that positively impact on pregnancy or neonatal outcomes, coupled with no national guidelines on weight gain in pregnancy and National Institute of Health and Care Excellence (NICE) guidance that was seen to be out-of-date, made it difficult for providers to know what services to commission and how to effectively evaluate current services.

*“Our NICE guidelines for weight monitoring, if you want the truth they are so woolly you could never evaluate it, because it doesn’t specifically say who to do what ... It is not a proactive guide in my opinion.” Participant 4.*

*“It would help if current NICE guidelines were appropriate … we were so looking forward to them coming out and … when they did they were very meek and all they were talking about was about myth-busting … These guidelines are totally out of date … they need updating and they need more teeth as well.” Participant 7.*

One respondent felt it also led to NHS trusts all developing their own thing, when a lot of time and effort could be saved with national level input.

#### An uphill struggle

Participants felt the public viewed being overweight as ‘normal’, due to increasing population prevalence. The media propagated image of healthy eating and physical activity being middle-class and too expensive for women from deprived communities also needs addressing to reduce inequitable access.

*“there is a perception I think that healthy eating and being physically active is quite a middle-class thing and I think that’s a real issue ... and that’s not helped by the media ... I know from experience when I’ve delivered sessions and it was about sugar and our children having sugar and she* [a mother] *was like I don’t want my child to be an effing snob by not having sugar.”* Participant 1.

Work commitments and employers not facilitating access to healthy lifestyle appointments also made it difficult for women to benefit from services. Finally, women wanting to lose weight often undertake it themselves rather than going to a healthcare professional for support to achieve their goal. Group interventions were especially felt to inhibit access for some women.

### Need for additional support

#### Educating women

There was a call for more pre-conception education, either through schools or a national campaign highlighting the risks to mother and child of being overweight at conception, to reduce the number of women with a raised BMI prior to pregnancy. Incorporating aspects such as weight maintenance and cooking skills into antenatal classes was also suggested.

*“ideally you don’t want them to go into pregnancy overweight … I think you start at school because they are potentially your mothers of the future.” Participant 4.*

*“ideally with women who are overweight it would be nice if they lost some weight before they got pregnant, which some of them do, but not all of them because some of them are oblivious!” Participant 2.*

#### Educating professionals

Training all healthcare professionals prior to registration was felt to be essential, so healthy eating is an integral part of the job from the start.

*“Ideally we should be starting with the student midwives in university and then the newly qualified midwives, so that actually, that message is from the start of their midwifery training. ... No, it’s not an extra, it’s not something that they learn afterwards, it’s part of their training.”* Participant 4.

Healthcare professionals who themselves were obese or struggled with their weight were seen to lack confidence to raise the topic with women. Training staff to understand behaviour change theory, personal motivators and to initiate conversations, including those who traditionally don’t have a public health role, was seen as crucial to achieve the ethos of Make Every Contact Count.

*“We’re looking at things like workforce development and … trying to train up parts of the workforce that perhaps wouldn’t have traditionally been … and sometimes people from different services have better relationships with families, we know that a lot of our housing officers for example, have good relationships with families.” Participant 13.*

Furthermore, service availability for pregnant women could be improved by training providers on the needs of pregnant women and how to incorporate them into existing adult services.

#### Maternity service changes

Many respondents wanted further maternal obesity services, either bespoke or the commissioning of programmes such as Slimming World for all pregnant women. Continuity of carer during pregnancy was also desired to assist with conversations and follow-up regarding healthy lifestyle. A desire for personalised trajectories for monitoring weight during pregnancy was also voiced, however this would require services in place for onward referral if women’s weight gain exceeded expectations. Better liaison between midwives and health visitors for women with a raised BMI to prevent weight gain between pregnancies was also called for.

*“I think, if we had a secure evidence base that enabled us to say, ‘this is a good trajectory for you’, … similar to ... customised growth charts for plotting a foetus, ... we could follow them ... But, also we’d need to know what to say if somebody’s growth exceeds; what to do, what to offer, where to refer them, how to help them”* Participant 7.

## Discussion

Healthy eating, weight management and physical activity services for all women were varied in nature from nothing or the provision of very basic information to structured weight management or physical activity programmes. Service provision for women with a BMI ≥ 25 kg/m^2^ was found to be minimal, particularly for physical activity interventions. The results regarding availability of services was consistent between the semi-structured interviews and the survey.

This study showed clear variation in maternal obesity service provision across England with a complete lack of accessible services in a number of areas, especially for women with a high BMI. Multiple respondents within a single LMS had differing awareness of service provision. Participants in the semi-structured interviews suggested this could have been due both to service inequity between different areas and also due to the constant commissioning and decommissioning of services making it difficult for practitioners to stay informed of service provision. It is essential therefore that healthcare professionals who have contact with women prior to pregnancy, during pregnancy and postpartum are informed of up-to-date local service provisions. Clearer national leadership is also required on the commissioning requirements around maternal healthy weight to help reduce geographical variation and the potential health inequalities that this may cause.

While numerous services had worked hard to involve service users in the development or update of services, very few reported being evaluated. The majority of those that had been evaluated had done so through internal audit. More needs to be done to formally evaluate services. This needs to include evaluation of the effective behaviour change components of the service through frameworks such as Michie et al. [25] who developed a taxonomy of behaviour change techniques used within interventions. This is in line with Public Health England who have recommended the application of an evidence based framework to ensure the embedding of appropriate behaviour change techniques into interventions [26] and have provided a list of the most effective behaviour change techniques to use in future weight management interventions [27]. A recent review of behaviour change techniques used in gestational weight management trials has found these techniques are currently poorly reported [28]. Future services need to more clearly elaborate on the behaviour change techniques incorporated within specific interventions, so that active components of interventions can be identified and more readily reproduced. There should also be a focus on the extent to which services are delivering evidence-based interventions as intended. Research has consistently shown that evidence translation is problematic [29] and calls have been made to utilise appropriate behaviour change techniques for health professional behaviour change [30]. More concise, clearer and directive national guidance would also enable existing services to be better evaluated for effectiveness. This could prevent local areas ‘re-inventing the wheel’ with a limited budget. NICE should therefore consider urgently reviewing the maternal obesity guidelines [13] so that they reflect up to date evidence.

The main barrier identified by participants to providing and commissioning healthy weight services for the childbearing population was the lack of, or inconsistency in, funding. Funding to public health budgets has seen significant cuts over recent years which has had significant effects on public health services across the board [31]. To tackle the increasing problem of maternal obesity it is therefore important that good practice is shared effectively when services have been evaluated robustly and found to improve maternal health outcomes. This is required to meet the vision of workstream 9 ‘improving prevention and population health’ within the maternity transformation programme to improve health by preventing poor outcomes and improve woman’s health before, during and after pregnancy [32].

Commissioners should consider implementing strategies to reduce the barriers identified through the interviews especially as these are likely to affect women living in deprived areas disproportionately to their more affluent counterparts. Initiatives such as de-centralising services into local areas with good transport links and childcare provision may help to facilitate women’s access, particularly for women from deprived areas. Making all services free at the point of use needs to be considered, alongside a proper economic evaluation to determine the cost effectiveness of such a strategy. These facilitators were also identified in a recent study addressing how lifestyle interventions could be tailored to improve access and ultimately outcomes for low socio-economic populations [33]. The importance of consulting the target population to better understand their service needs and to ensure services developed are relevant and appropriate cannot be overlooked [26].

National educational resources should be developed to educate women and healthcare providers around healthy maternal weight prior to pregnancy, during pregnancy and in the postpartum period. This is necessary both to maximise the use of available services and to ensure consistent reliable messaging around maternal healthy weight.

Previous research has shown regional differences in the rate of maternal obesity and clear evidence of health inequalities in relation to a higher incidence of maternal obesity and its complications among women from Black and Ethnic Minority (BME) backgrounds and those who are socially deprived [2, 5, 34]. It is therefore important to consider sociodemographic predictors of maternal obesity and its complications in any future research to ensure equitable service provision.

### Limitations of this study

This research was enhanced by the participants in both phases of the study representing a wide variety of occupations and including both commissioners and providers. Survey respondents were also geographically well dispersed across England and the interviewees covered numerous councils and NHS trusts. However, like any other survey this represents a self-selected sample of respondents. The fact that only 23 of the 44 LMSs provided a response to the survey was also a limitation of the project. However complementary approaches in this research present findings which indicate discrepancies in service provision from various key stakeholders’ perspectives. It also highlighted the need for rigorous evaluation of existing services and equitable provision of services particularly before and after pregnancy.

## Conclusion

Healthy weight service provision varies in different geographical areas across England. It is therefore important to ensure all healthcare workers are aware of related service provisions. More maternity healthy weight services are needed with an emphasis on physical activity. Service provision and access also needs to be encouraged prior to pregnancy and in the postnatal period, particularly for those with a raised BMI. To ensure these services are fit for purpose more robust evaluation is required. Finally, healthcare providers should be aware of the existing services to encourage their uses. They also require clear guidance and training to support pregnant women achieve a healthy weight gain.

## Supplementary information

**Additional file 1.** Provision of maternal healthy weight services survey.

**Additional file 2.** Stakeholder interview schedule.

**Additional file 3.** Maternal Healthy Weight Service Provision in England Infographic.

## Data Availability

The datasets used and/or analysed during the current study are available from the corresponding author on reasonable request.

## Abbreviations

BMI: Body Mass Index
LMS: Local Maternity Systems
NHS: National Health Service
UK: United Kingdom
PHE: Public Health England
HENRY: Health, Exercise and Nutrition for the Really Young
NICE: National Institute of Health and Care Excellence
WS9: Workstream 9
