# Supplementary material for: Healthy weight services in England before, during and after pregnancy: a mixed methods approach
Source: BMC Health Serv Res. 2020 Jun 22;20:572. doi: 10.1186/s12913-020-05440-x (PMC7310438; doi:10.1186/s12913-020-05440-x)
Supplement: Supplementary file 1 — Additional file 1. Provision of maternal healthy weight services survey. [file 12913_2020_5440_MOESM1_ESM.docx]

**Additional File 1. Provision of maternal healthy weight services survey**

1. Are you a commissioner or a provider?
2. What type of service do you commission/provide?
   1. Maternity services (direct to question 3 to 5 and NOT Qu 6)
   2. Sexual health and contraceptive services (all other options directed to Qu 6)
   3. Post termination/miscarriage services
   4. Fertility services
   5. Diabetes services
   6. Mental health services
   7. Epilepsy services
   8. Stop smoking services
   9. Primary care
   10. Health visiting services
   11. Local authority
   12. Dietetics
   13. Healthy lifestyle services
   14. Other - please state
3. Which of the following bests describes your unit:
   1. Obstetric Unit
   2. Alongside Midwifery Unit
   3. Free Standing Midwifery Unit
   4. Other - please specify
4. How many births does your unit undertake each year?
5. Do you link with local health visitor or general practice nurses to encourage healthy lifestyle in the women that you have contact with?
6. How many women does your service see each year?
7. Which Local Maternity System are you in?
   - - Northumberland, Tyne and Wear
     - West, North and East Cumbria
     - Durham, Darlington, Tees, Hambleton, Richmondshire and Whitby
     - Lancashire and South Cumbria
     - West Yorkshire
     - Coast, Humber and Vale
     - Greater Manchester
     - Cheshire and Merseyside
     - South Yorkshire and Bassetlaw
     - Staffordshire
     - Shropshire and Telford and Wrekin
     - Derbyshire
     - Lincolnshire
     - Nottinghamshire
     - Leicester, Leicestershire and Rutland
     - The Black Country
     - Birmingham and Solihull
     - Coventry and Warwickshire
     - Herefordshire and Worcestershire
     - Northamptonshire
     - Cambridgeshire and Peterborough
     - Norfolk and Waveney
     - Suffolk and North East Essex
     - Milton Keynes, Bedfordshire and Luton
     - Hertfordshire and West Essex
     - Mid and South Essex
     - North West London
     - North Central London
     - North East London
     - South East London
     - South West London
     - Kent and Medway
     - Sussex and East Surrey
     - Frimley Health
     - Surrey Heartlands
     - Cornwall and the Isles of Scilly
     - Devon
     - Somerset
     - Bristol, North Somerset and South Gloucestershire
     - Bath, Swindon and Wiltshire
     - Dorset
     - Hampshire and the Isle of Wight
     - Gloucestershire
     - Buckinghamshire, Oxfordshire and Berkshire West
8. Are there any services universally available to all women attending your service to encourage them to eat a healthy diet;
   - prior to pregnancy? Y/N
   - during pregnancy? Y/N
   - up to 1 year postpartum? Y/N

IF YES

- - - Could you please describe these? (what is provided, to whom, by whom, at what stage of the childbearing cycle)
      - Do these services take a family approach? Y/N
      - If yes, please describe.
      - Have these services been assessed or evaluated? Y/N
    - If yes, could you describe the results or provide a reference if possible?
    - If yes, were there any assessments in terms of how the different components of the service (e.g. the behaviour change techniques used) might change dietary behaviour?
    - Have service users been involved in the development of services? Y/N
    - If yes, please describe their role

1. Are there any services universally available to all women attending your service to encourage them to undertake physical activity;
   - - prior to pregnancy? Y/N
     - during pregnancy? Y/N
     - up to 1 year postpartum? Y/N

IF YES

- - - Could you please describe these? (what is provided, to whom, by whom, at what stage of the childbearing cycle)
      - Do these services take a family approach? Y/N
      - If yes, please describe.
      - Have these services been assessed or evaluated? Y/N
    - If yes, could you describe the results or provide a reference if possible?
    - If yes, were there any assessments in terms of how the different components of the service (e.g. the behaviour change techniques used) might change physical activity behaviours?
    - Have service users been involved in the development of services? Y/N
    - If yes, please describe their role

1. Do any have services universally available to all women attending your service to help them to manage their weight;
   - prior to pregnancy? Y/N
   - during pregnancy? Y/N
   - up to 1 year postpartum? Y/N

IF YES

- - - Could you please describe these? (what is provided, to whom, by whom, at what stage of the childbearing cycle)
      - Do these services take a family approach? Y/N
      - If yes, please describe.
      - Have these services been assessed or evaluated? Y/N
    - If yes, could you describe the results or provide a reference if possible?
    - If yes, were there any assessments in terms of how the different components of the service (e.g. the behaviour change techniques used) might change healthy weight behaviours?
    - Have service users been involved in the development of services? Y/N
    - If yes, please describe their role

1. What is the approximate percentage of women with a BMI≥25kg/m² who book into your service each year?
2. What is the approximate percentage of women with a BMI≥30kg/m² who book into your service each year?
3. Do you have additional services available at your unit to encourage women with a BMI ≥25kg/m² to manage their weight and/or eat a healthy diet;
   - prior to pregnancy? Y/N
   - during pregnancy? Y/N
   - up to 1 year postpartum? Y/N

IF YES

- - - Could you please describe these? (what is provided, to whom, by whom, at what stage of the childbearing cycle)
      - Do these services take a family approach? Y/N
      - If yes, please describe.
      - Have these services been assessed or evaluated? Y/N
    - If yes, could you describe the results or provide a reference if possible?
    - If yes, were there any assessments in terms of how the different components of the service (e.g. the behaviour change techniques used) might change healthy weight behaviours?
    - Have service users been involved in the development of services? Y/N
    - If yes, please describe their role

1. Do you have additional services available at your unit to encourage women with a BMI ≥25kg/m² to undertake physical activity;
   - prior to pregnancy? Y/N
   - during pregnancy? Y/N
   - up to 1 year postpartum? Y/N

IF YES

- - - Could you please describe these? (what is provided, to whom, by whom, at what stage of the childbearing cycle)
      - Do these services take a family approach? Y/N
      - If yes, please describe.
      - Have these services been assessed or evaluated? Y/N
    - If yes, could you describe the results or provide a reference if possible?
    - If yes, were there any assessments in terms of how the different components of the service (e.g. the behaviour change techniques used) might change physical activity behaviours?
    - Have service users been involved in the development of services? Y/N
    - If yes, please describe their role

1. Are there any third sector / voluntary organisations in your area that provide additional support or services to pregnant women to encourage healthy weight management or physical activity before, during or after pregnancy?
   - If so could you briefly describe these?
2. Are you aware of any other models of good practice regarding the encouragement of;
   - a healthy weight for women before pregnancy? (Y/N)
     - If yes, please describe or give a reference.
   - a healthy weight gain during pregnancy? (Y/N)
     - If yes, please describe or give a reference.
   - a healthy weight for women after pregnancy? (Y/N)
     - If yes, please describe or give a reference.
3. Do you provide any other interventions or support to promote healthy diet, physical activity or healthy weight management before pregnancy, during pregnancy or up to 1 year postpartum? Please specify (e.g. make every contact count, links to leisure centres/ walking programmes).
