# Supplementary material for: Healthy weight services in England before, during and after pregnancy: a mixed methods approach
Source: BMC Health Serv Res. 2020 Jun 22;20:572. doi: 10.1186/s12913-020-05440-x (PMC7310438; doi:10.1186/s12913-020-05440-x)
Supplement: Supplementary file 2 — Additional file 2. Stakeholder interview schedule. [file 12913_2020_5440_MOESM2_ESM.docx]

**Additional File 2. Stakeholder interview schedule**

We are very grateful to you for sparing time to take part in this interview today. There are two sections to the interview, the first part is to explore maternal healthy weight service provision locally and the second part to explore how you think effective maternity weight interventions could best be evaluated.

The interview will be recorded and then written up word for word to make it easier for the researchers to evaluate what has been said afterwards.

1. We want to ask about any services you have for women **prior to pregnancy**, **during pregnancy** or **up to one year postpartum** that encourage women to:

- eat a healthy diet
- undertake physical activity
- achieve a healthy weight
  - - Do you have any **universal** services available locally covering these areas?
    - If yes, could you please describe these? (what is provided, to whom, by whom, at what stage of the childbearing cycle)
    - Do these services take a family approach?
    - Have these services been assessed or evaluated?
      - If yes, could you describe the results or provide a reference if possible?
      - If yes, were there any assessments in terms of how the different components of the service (the behaviour change techniques used) might change weight management behaviours?
    - Have service users been involved in the development of services?
      - If yes please describe their role

1. What is the approximate percentage of women with a BMI≥25kg/m² who book into your service each year?
2. What is the approximate percentage of women with a BMI≥30kg/m² who book into your service each year?
3. Again with regards to services for women **prior to pregnancy**, **during pregnancy** or **up to one year postpartum** that encourage women to

- eat a healthy diet
- undertake physical activity
- achieve a healthy weight
- Do you have additional services available locally for women with a BMI ≥25kg/m²?
  - - If yes, could you please describe these? (what is provided, to whom, by whom, at what stage of the childbearing cycle)
    - Do these services take a family approach?
    - Have these services been assessed or evaluated?
      - If yes, could you describe the results or provide a reference if possible?
      - If yes, were there any assessments in terms of how the different components of the service (the behaviour change techniques used) might change weight management behaviours?
    - Have service users been involved in the development of these services?
      - If yes please describe their role

1. What factors do you think are facilitators locally for providing and accessing healthy weight management services to women before, during or after pregnancy?
2. What factors do you think are barriers locally for providing and accessing healthy weight management services to women before, during or after pregnancy?
3. Are there any areas of service provision related to healthy eating and physical activity that you would like to see improved?
4. Are there are any further resources that could assist you in supporting healthy weight and physical activity before/ during or after pregnancy?
5. Do you have any staff training about healthy weight and physical activity for women prior to, during or after pregnancy?
   - If yes, when is this provided, to who, what is covered, does it include behaviour change techniques, what format (ie face-to-face, web-based etc)?
6. Are there any third sector / voluntary organisations that provide additional support or services to pregnant women to encourage healthy weight management or physical activity before, during or after pregnancy in your area?
   - If so could you briefly describe these?
7. Do you have any further comments you would like to make about the services in general or how best to support pregnant women to live healthy lifestyles?

The second part of this interview is to explore your views on how best to assess the effectiveness of healthy lifestyle interventions.

1. Do you think there is a need to assess behaviour change techniques used in interventions/ support services aimed at
   - improving healthy weight prior to pregnancy?
   - preventing excessive weight gain during pregnancy or
   - maintaining a healthy weight status following a pregnancy
   - If yes, what behaviour change techniques should be included?
   - Are you aware of any outcome measures for behaviour change for interventions aimed at improving healthy weight / preventing excessive weight gain?
2. Which adverse health outcomes for high risk women and their babies do you consider it important to reduce through healthy lifestyle interventions?
3. Which outcomes do you think are important to consider from a cost point of view:
   - prior to pregnancy?
   - during the antenatal period?
   - during delivery?
   - during the immediate postpartum period?
   - from birth to one year postpartum?
4. How best could a reduction in women experiencing health risks due to their weight status during pregnancies be measured?
5. How could services be assessed for their accessibility?
6. How could the uptake of services be assessed?
7. How could services be assessed for their effectiveness at reducing inequalities?
8. How could adherence to current NICE guidelines be assessed?
9. Do you have any other comments you wish to make?

**Thank you again for taking part, we really appreciate it.**
