# Supplementary figures and images for: Healthy weight services in England before, during and after pregnancy: a mixed methods approach
Source: BMC Health Serv Res. 2020 Jun 22;20:572. doi: 10.1186/s12913-020-05440-x (PMC7310438; doi:10.1186/s12913-020-05440-x)

**Additional File 3. Maternal Healthy Weight Service Provision in England Infographic**


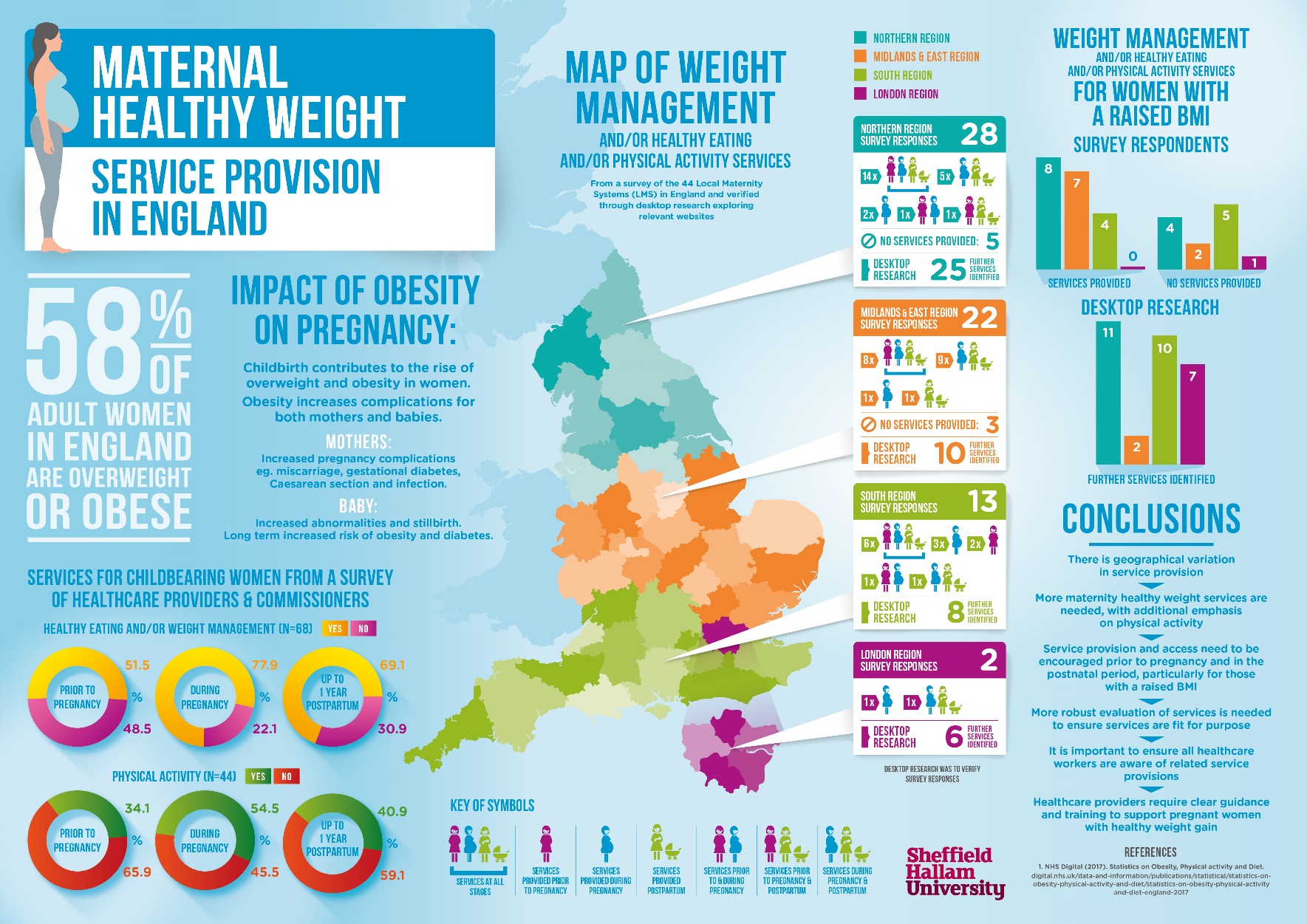

Supplement: Supplementary file 3 — Additional file 3. Maternal Healthy Weight Service Provision in England Infographic. [file 12913_2020_5440_MOESM3_ESM.docx]
